# Pathogenicity and virulence of *Burkholderia pseudomallei*

**DOI:** 10.1080/21505594.2022.2139063

**Published:** 2022-11-03

**Authors:** Nicole M. Bzdyl, Clare L. Moran, Justine Bendo, Mitali Sarkar-Tyson

**Affiliations:** The Marshall Centre for Infectious Diseases Research and Training, School of Biomedical Sciences, University of Western Australia, Perth, Western Australia, Australia

**Keywords:** Virulence, melioidosis, pathogenesis, *Burkholderia pseudomallei*, actin tails, multinucleated giant cells

## Abstract

The soil saprophyte, *Burkholderia pseudomallei*, is the causative agent of melioidosis, a disease endemic in South East Asia and northern Australia. Exposure to *B. pseudomallei* by either inhalation or inoculation can lead to severe disease. *B. pseudomallei* rapidly shifts from an environmental organism to an aggressive intracellular pathogen capable of rapidly spreading around the body. The expression of multiple virulence factors at every stage of intracellular infection allows for rapid progression of infection. Following invasion or phagocytosis, *B. pseudomallei* resists host-cell killing mechanisms in the phagosome, followed by escape using the type III secretion system. Several secreted virulence factors manipulate the host cell, while bacterial cells undergo a shift in energy metabolism allowing for overwhelming intracellular replication. Polymerisation of host cell actin into “actin tails” propels *B. pseudomallei* to the membranes of host cells where the type VI secretion system fuses host cells into multinucleated giant cells (MNGCs) to facilitate cell-to-cell dissemination. This review describes the various mechanisms used by *B. pseudomallei* to survive within cells.

## GRAPHICAL ABSTRACT

**Figure:**
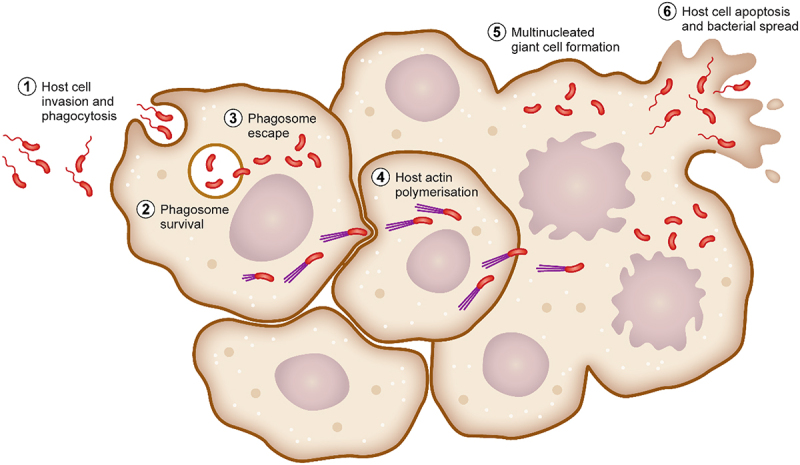

## Introduction

The soil saprophyte, *Burkholderia pseudomallei*, is the causative agent of melioidosis, a multifaceted disease endemic to the tropical belt, with hotspots in South East Asia and northern Australia [1,2]. Despite leading a typically environmental lifestyle, *B. pseudomallei* is able to infect exposed humans and animals causing disease and mortality [3–5]. Diabetes is the leading risk factor for severe illness, with in excess of 40% of melioidosis sufferers having diabetes, or being diagnosed with diabetes at the time of hospital presentation [6]. The International Diabetes Federation (IDF) estimates that in South East Asia and the western Pacific, around 10% of the adult population lives with diabetes, with over half of those being undiagnosed [7,8]. These rates are predicted to steadily increase over the next two decades. In addition, international travel has grown exponentially in recent years, with 120 million international arrivals into South East Asia alone in 2017 [9]. This highlights a disproportionately susceptible population living in highly endemic areas, as well as millions of regional visitors at risk of exposure to *B. pseudomallei*. This review aims to highlight what is known about the pathogenicity and virulence of *B. pseudomallei*.

## The *Burkholderia* sensu stricto group

The *Burkholderia* sensu lato (*Burkholderia* sl.) group, formerly classified as the genus *Burkholderia* and previous to that, *Pseudomonas*, has recently undergone a taxonomical reshuffle partly due to the increased availability of genome sequences from the *Burkholderia* species [10,11]. *Burkholderia* sl. encompasses six genera: *Burkholderia* sensu stricto (*Burkholderia* ss.), *Paraburkholderia*, *Trinickia*, *Robbsia*, *Mycetohabitans*, and *Caballeronia* [11]. Most species of *Burkholderia* sl. are found in the environment, particularly the soil, but can occupy many ecological niches such as the rhizosphere, plants, and root nodules [12]. Several species are capable of causing disease in plants such as onion rot (*T. caryophylii*), leaf spot and stripe disease (*R. andropogonis*), and rot of rice grains (*B. glumae*) [13–16]. The *Burkholderia* ss. group consists of predominantly opportunistic pathogens, which fall into one of two species groups: *B. cepacia* complex (BCC) or *B. pseudomallei* complex (BPC) [17,18]. The *Burkholderia cepacia* complex consists of 22 closely related species that commonly infect immunocompromised and cystic fibrosis patients, with the most notable of these being *B. cenocepacia* and *B. multivorans* [19–21]. Species of BCC commonly cause chronic pulmonary infection in cystic fibrosis, patients as well as nosocomial infection in immunocompromised individuals [22,23]. The most severe presentation of disease includes rapid and uncontrolled deterioration of patients with onset of septicaemia and necrotising pneumonia, commonly referred to as “cepacia syndrome” [24]. As with most *Burkholderia* species, BCCs are intrinsically multi-drug resistant and capable of long-term persistence in the lung due to the establishment of *in vivo* biofilms [25]. Multi-week courses of at least two intravenous antibiotics, such as tobramycin, meropenem, or ceftazidime, are commonly prescribed to cystic fibrosis sufferers with pulmonary exacerbations [23,26].

The *Burkholderia pseudomallei* complex (BPC) now contains eight highly related species: *B. pseudomallei*, *B. mallei*, *B. thailandensis*, *B. humptydooensis, B. oklahomensis*, *B. singularis*, *B. mayonis*, and *B. savannae* [18,27]. The most studied members of the BPC are *Burkholderia pseudomallei* and *Burkholderia mallei*, which cause melioidosis and glanders, respectively [28,29]. *Burkholderia thailandensis*, a close relative of *B. pseudomallei*, rarely causes human disease and is largely considered avirulent [30,31]. *B. pseudomallei* and *B. mallei* are classified as Tier 1 Select Agents by the Center for Disease Control and Prevention (CDC) with international guidelines recommending handling within a class II biosafety cabinet (BSC) in a biosafety level 3 (BSL3) facility, making genetic manipulation and characterisation difficult [32]. *Burkholderia mallei* is the causative agent of both glanders (a naso-pulmonary syndrome) and farcy (cutaneous infection), which not only primarily infects solipeds such as horses and donkeys but can also cause fatal human disease upon exposure [29]. Human cases are sporadic and most commonly associated with people working in proximity to infected animals [33]. Glanders is endemic in parts of the Middle East, Asia, Africa, and Central and South America [34]. Interestingly, *B. mallei* is the only *Burkholderia* species that is an obligate intracellular pathogen and can cause fulminant disease in less than a week if acute symptoms manifest [35]. It is thought that *B. mallei* evolved from a single clone of *B. pseudomallei* after the loss of over 1000 genes. The remaining genes share >90% sequence homology with *B. pseudomallei*, supporting this claim [36,37].

## Melioidosis

Melioidosis is a multi-syndrome illness, first described over a century ago by Alfred Whitmore in Rangoon, Myanmar [28], and for many years, was referred to as Whitmore’s Disease. Although predominantly a tropical illness [38], importation of cases from returned travellers has also been documented [39–41]. Overall, both cases and deaths are thought to be highly under-reported, primarily due to lack of disease awareness, misdiagnosis, and lack of seeking healthcare. As such, an accurate global burden is difficult to compile [2,6,38,42]. Modelling has estimated that there are around 165,000 cases of human melioidosis per annum globally, with up to 89,000 thousand deaths [2]. The highly endemic hotspots of northern Australia and north east Thailand have reported incidence rates of up to 50 cases per 100,000 people [6,43]. Surrounding countries in South East Asia, such as Laos, Cambodia, and Vietnam, also report high levels of incidence [38,44,45]. In north east Thailand, melioidosis is the third most common cause of infectious disease death behind only AIDS and tuberculosis, with a mortality rate of around 40% [6]. Mortality rates are lower in northern Australia at 10%, probably due to increased public disease awareness and better access to tertiary medical care [46]. A systematic review of all melioidosis case reports, conducted in 2019, allowed for modelling to determine the global burden of melioidosis. The burden of symptomatic melioidosis worldwide is 4.6 million DALYs (Disability-adjusted life-year) annually, of which over 98.9% is attributed to years of life lost (YLL) [47]. This burden from melioidosis exceeds what is reported for more prominent diseases such as dengue fever (1.95 million DALYs), leishmaniasis (0.7 million DALYs), and schistosomiasis (1.6 million DALYs) [48]. Taking into consideration both global modelling and local incidence, melioidosis is highly prevalent worldwide and is a significant cause of death in regions where it is endemic.

Melioidosis appears to be a predominantly opportunistic infection, with only 20% of patients presenting with no known risk factors. The main risk factors for melioidosis include diabetes, hazardous alcohol use, and chronic lung or renal disease [6,43,49]. In north east Thailand, the incidence rate of melioidosis for non-diabetics is 6.8 per 100,000, while incidence in diabetics is 145.7 per 100,000 and 84.4 per 100,000 in undiagnosed diabetics [6].

Melioidosis has been referred to as the “great mimicker” due to the wide variety of clinical symptoms that can be observed in patients presenting to hospitals around the globe [1,50,51]. Pneumonia is the predominant clinical presentation of melioidosis and can appear in both acute and chronic cases [42,52], this is followed by non-healing skin lesions [43,53,54]. The breadth of clinical manifestations can lead to difficulty in prompt disease diagnosis, particularly in tropical regions that are also endemic for diseases such as malaria and tuberculosis, and as a result, melioidosis cases are commonly misdiagnosed [39,54,55].

Treatment consists of two phases: an intravenous intensive initial phase followed by an oral eradication therapy [56]. A minimum of 14 days of intravenous therapy is required with either ceftazidime or meropenem [57,58]. Following the intensive phase of therapy, a prolonged treatment with orally administered trimethoprim-sulfamethoxazole is required to allow for the successful eradication of infection and to prevent infection recrudescence or relapse [57,59–62].

### Burkholderia pseudomallei

*B. pseudomallei* possesses one of the largest bacterial genomes (7.2 Mb), which is shared over two chromosomes and contains an extensive arsenal of virulence determinants, which enable it to both survive a range of harsh environments and cause a multitude of clinical presentations of disease [63]. Due to its environmental niche, *B. pseudomallei* is intrinsically resistant to many host factors, anti-bacterial agents, and antibiotics [64]. Ultimately, this not only makes *B. pseudomallei* infection difficult to treat and eradicate but also makes elucidating the pathogenesis and the roles of virulence factors extremely challenging due to the many redundancies within its genome.

Following exposure to the bacterium, *B. pseudomallei* is able to attach to and invade host cells, as well as being phagocytosed into phagosomes by macrophages and neutrophils. *B. pseudomallei* resists host-cell killing mechanisms within the phagosome and then rapidly escapes into the cytoplasm. Rapid intracellular proliferation of bacteria then occurs as well as changes to primary bacterial energy production to allow for utilisation of available nutrients as well as subversion of the host through secretion of cytopathic toxins. *B. pseudomallei* then polymerises host-cell actin to propel itself to the host cell membrane where it induces host cell fusion into multinucleated giant cells (MNGCs), allowing it to spread throughout the body.

The host response to *B. pseudomallei* infection has recently been reviewed by Chomkatekaew et al. [65]. Hence, this review will focus on the intracellular lifestyle of *B. pseudomallei* and describe the key determinants that affect the ability of the bacterium to survive in cells.

### Adhesion to and invasion of host cells

**Figure 1.**
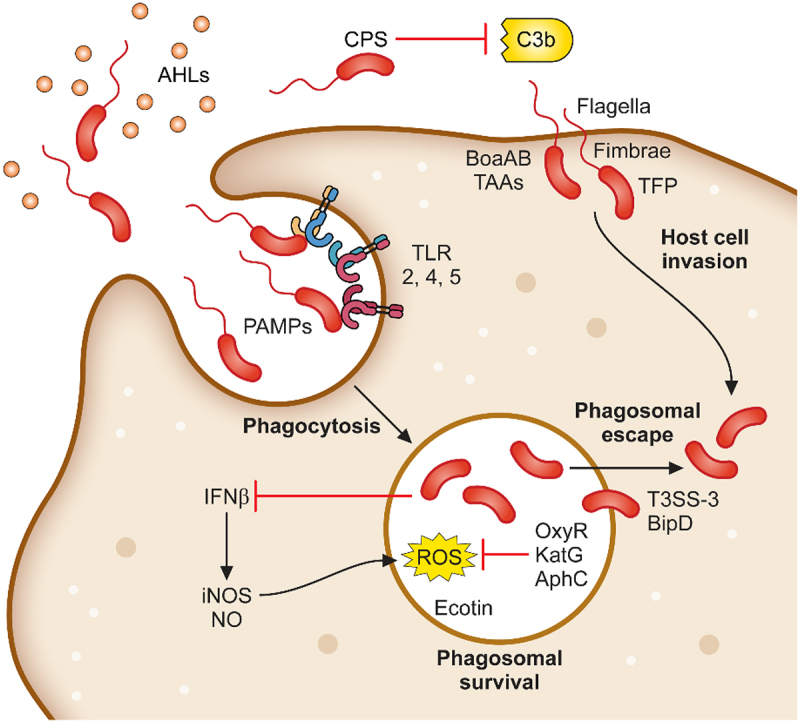
Determinants required for *B. pseudomallei* attachment, invasion and phagosomal survival. *B. pseudomallei* uses a variety of mechanisms to initiate infection of host cells. Organelles and proteins such as the flagella, fimbriae, TFP and TAAs are used to attach to and directly infect host cells. In addition, *B. pseudomallei* is phagocytosed by macrophages. Following phagocytosis, *B. pseudomallei* induces expression of a plethora of proteins involved in phagosomal survival which as OxyR, KatG and AphC which protects against reactive oxygen species. Phagosomal escape is subsequently mediated by the type III secretion system which allows for rapid escape into the cytoplasm.

*B. pseudomallei* is able to infect virtually every cell type within a host [66]. The bacterium is recognised by professional phagocytes, which subsequently phagocytose bacteria into a phagosome. *B. pseudomallei* also encodes for filamentous appendages and proteins, which facilitates tight binding to non-phagocytic cells and then subsequent invasion into the cell (Figure 1). Exposure to *B. pseudomallei* can occur via inhalation, percutaneous inoculation, or accidental ingestion [67]. It is thought that inhalation and incidental inoculation during occupational or recreational activity are the most frequent routes of bacterial acquisition. Breach of the epidermal layer may permit penetration by *B. pseudomallei* or contaminated matter (such as soil or surface water) into dermal tissue, extracellular matrices, endothelial cells, the bloodstream, and sufficiently traumatic, deeper organ tissues [68–70]. Alternatively, after inhalation of aerosolised *B. pseudomallei*, bacterial cells are deposited along the upper and lower respiratory tract according to the droplet size as demonstrated by the BALB/c murine melioidosis model [71], with bacteria in smaller droplets (1 µm) observed to colonise the lower respiratory epithelium, while those in larger droplets (12 µm) are primarily associated with nasal mucosa and nasal-associated lymphoid tissue (NALT) [72]. Regardless of the initial colonisation site, *B. pseudomallei* infection rapidly spreads around the body with colonisation seen in distal organs within 72 hours [72,73]. After exposure, *B. pseudomallei* establishes physical contact with both phagocytic and non-phagocytic host cells and facilitates phagocytosis or cell invasion to establish an intracellular replicative niche.

## Pili

Pili, made up of pilin proteins, are filamentous appendages used by bacteria to mediate the initial stages of host cell adherence. Pili are able to retract and extend through filament polymerisation and depolymerisation to move along surfaces and come into close contact with host cells [74]. A total of 13 pilin-like clusters are encoded in the prototype K96243 strain of *B. pseudomallei* [63]. Deletion of *pilA*, which encodes a class A type IV pilus (TFP) in strain K96243, resulted in reduced adherence or invasion compared to the parental strain in three human epithelial cell lines, A549, BEAs2-B, and RPMI-2650 [75]. Infection of the nematode, *Caenorhabditis elegans*, with the *pilA* mutant resulted in an 18-hour delay to death of 58 hours compared to 40 hours by the wild-type strain. Interestingly, this mutant showed no difference in its ability to cause disease in BALB/c mice compared to the wild-type strain by the intraperitoneal route, but a delay in death was observed when challenged by the intranasal route, with a lower bacterial challenge dose [75]. In contrast, a *pilA* mutant generated from an alternative strain, *B. pseudomallei* strain 08, did not exhibit the same reduction in cellular adhesion or invasion against ME-180 human epithelial cells as was described by Essex-Lopresti et al. during their investigation of the K96243 strain. Rather, *pilA* was required by strain 08 for microcolony formation but only when grown at the lower temperature of 27°C [76]. Deletion of a class B TFP cluster (*BPSS2185-2198*) resulted in attenuation in BALB/c mice when challenged intranasally [77]. Further studies, where just the pilus subunit, *BPSS2185*, was deleted, resulted in a 50% decrease in adherence to A549 human lung epithelial cells [78]. To fully understand the mechanism behind pilin-mediated attachment and/or adherence, further studies into the other pilin-like clusters are required.

## Fimbriae

Another filamentous structure present on the outer surface of bacteria is the type I fimbriae, which bind to D-mannosylated sugar residues located on the cell surface glycoprotein receptors, expressed by intestinal epithelium and macrophages, thus facilitating internalisation of bacteria during intestinal infection [79]. Deletion of the type I fimbriae in *B. pseudomallei*, *fimA*, resulted in a decrease in adherence of *B. pseudomallei* to both human and murine intestinal epithelial cells, as well as significantly decreased plaque formation. The mutant strain, *ΔfimA* was also required for full virulence in a chronic intestinal model of melioidosis, with 90% survival after 35 days [80].

## Autotransporters

Autotransporters (ATs) are outer membrane-anchored proteins that have been implicated in multiple pathogenesis pathways and can exist as monomeric (classical ATs) or trimeric structures (trimeric autotransporter adhesins, TAAs). *B. pseudomallei* encodes eleven ATs, two classical ATs and nine TAAs [81]. Two *B. pseudomallei* TAAs, BoaA (BPSS0796) and BoaB (BPSL1705), act as adhesins, with deletion of *boaA* and *boaB* showing a decrease in adherence to A549, Hep2, and NHBE human respiratory epithelial cells, but only deletion of both TAAs in conjunction demonstrated a decrease in bacterial survival in J774A.1 murine macrophages [82]. Six of the other encoded TAAs, *bpaA, bpaB, bpaC, bpaD, bpaE,* and *bpaF* play a role in either adherence to or invasion of A549 human lung epithelial cells [83]. In the BALB/c mouse infection model, deletion of *bpaC* did not change the LD_50_ when challenged via an aerosol route of infection [84]. However, previous studies showed a decrease in dissemination of *ΔbpaC* to the liver at 48 hours post-infection when challenged intranasally, suggesting a role during the early stages of *in vivo* infection [83]. Another classical AT, BcaA, is involved in the invasion of non-phagocytic cells as a BcaA null-mutant exhibits a 50% reduction in invasive capacity of A549 human lung epithelial cells but exhibits no differences in phagocytic cells compared to the wild-type [85].

## Flagella

The flagellum of a bacterium not only allows for movement through the environment, but in many species, it is required for invasion of host cells. The *B. pseudomallei* flagellin subunit is encoded by *fliC* (*BPSL3319*). Deletion or inactivation of this gene results in a non-motile strain [86–89]. Initial studies to identify protective antigens demonstrated that passive immunisation with anti-flagellin IgG provided 80% protection against an intraperitoneal challenge with 10^4^ CFU of *B. pseudomallei* in a diabetic rat model [90]. Interestingly, disruption of the flagellin gene (via Tn*5* insertion) did not affect the virulence of *B. pseudomallei* mutant strains, MM35 and MM36, in the Syrian hamster and diabetic rat models of infection, leading to the hypothesis that the flagellum is not a virulence determinant per se but rather a protective antigen [88]. This was verified by another study where challenge of BALB/c mice by the intraperitoneal route with an alternative *B. pseudomallei* flagellin mutant also demonstrated no change in LD_50_ [89]. In contrast, deletion of *fliC* from the KHW strain of *B. pseudomallei* resulted in no attenuation during A549 human epithelial cell or *C. elegans* infection, but the *fliC* mutant did exhibit attenuation of infection in mice when challenged by the intranasal route, with decreased colonisation and mortality observed [86]. This lack of *in vitro* attenuation shown by Chua et al. was attributed to the use of a centrifugation step during experimental procedures, as omitting this step resulted in decreased initial intracellular counts in both RAW264.7 murine macrophages and A549 human epithelial cells [87], indicating that the flagellum is necessary for cell invasion and initiation of the intracellular lifecycle. The *B. pseudomallei* MM35-mutant strain lacking flagellum was also unable to attach to trophozoites of *Acanthamoeba astronyxis* [91], postulating that the flagellum is likely to be important in the very early stages of adhesion.

A predominant number (88%) of Australian *B. pseudomallei* isolates encode a *B. thailandensis*-like flagellum and chemotaxis biosynthesis (BTFC) gene cluster. This BTFC cluster was replaced through horizontal gene transfer with a *Yersinia*-like fimbriae (YLF) cluster, with YLF *B. pseudomallei* isolates dominating in South East Asia (98% of isolates) [92]. The BTFC *fla2* locus encodes laterally positioned flagella that is used by *B. thailandensis* and BTFC-*B. pseudomallei* strains to rapidly move intracellularly independent of host-actin polymerisation [93,94]. Although deletion of *fla2* does not affect swimming motility in *B. thailandensis* E264 [94], whether the BTFC locus provides a virulence advantage over the YLF locus is yet unknown.

The flagellin (FliC) of *B. pseudomallei* is also involved in recognition by toll-like receptors (TLR) which are crucial for the host detection of pathogens and stimulation of the innate immune system. Purified FliC is recognised by TLR5 receptors and on stimulation of HEK-Blue™-hTLR5 and THP1-Dual™, cells activate NF-κB, in a TLR5-dependent manner, although this stimulation is not at the same level as purified flagellin from *Salmonella* Typhimurium [95]. Amemiya et al. recently demonstrated that TLR5 recognises not only purified flagellin, but also the assembled filamentous flagella attached to *B. pseudomallei*, and deletion of *fliC* results in an elimination of TLR5 activation [96]. These observations warrant further investigation into the activation of TRL5 by the flagella/flagellin, since ways to subvert this would be advantageous to the host.

## Polysaccharides in *B. pseudomallei*

*B. pseudomallei* encodes numerous polysaccharide biosynthesis clusters, some of which have been well characterised. The nomenclatures of the various polysaccharide clusters in the literature can be confusing, as such Table 1 shows each polysaccharide cluster found in *B. pseudomallei* and the various nomenclatures used to describe them in the literature. This review will use the nomenclature proposed by Reckseidler-Zeneto et al. for the capsular polysaccharides [97] and Perry et al. for the *O*-antigen polysaccharides [98]. An exopolysaccharide of the structure [-3)-2-*O*-acetyl-*β*-D-Gal*p*-(1-4)-*α*-D-Gal*p*-(1-3)-*β*-D-Galp-(1-5)-*β*-D-KDO*p*-(2-] has also been identified in *B. pseudomallei,* but its precise role in virulence is yet to be elucidated [105,106].

**Table 1.** Polysaccharides produced by *B. pseudomallei*.

	Gene cluster (K96243)	Polysaccharide structure	Reference	Alternative name (reference)
Capsular polysaccharide (CPS)				
CPS I (main capsule type)	*BPSL2787-BPSL2810*	[-3)-2-*O*-acetyl-6-deoxy-*β*-D-*manno*-hepto-pyranose-(1-] 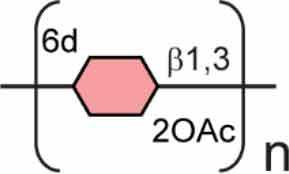	[97–102]	Type I *O*-PS [103] PS II [104]
CPS II	*BPSS0417-BPSS0429*	Not confirmed	[97]	Type III *O*-PS [103]
CPS III	*BPSS1825-BPSS1834*	Not confirmed	[97]	Type IV *O*-PS [103]
CPS IV	*BPSL2769-BPSL2785*	Not confirmed	[97]	
Lipopolysaccharide *O* Antigen (*O*-PS)				
Type II *O*-PS	*BPSL2672-BPSL2688*	[-3)-*β*-D-glucopyranose-(1-3)-6-deoxy-*α*-L-talo-pyranose-(1-] 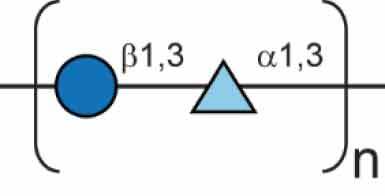	[98]	PS I [104] Type II *O*-PS [103]
Exopolysaccharide (EPS)				
EPS		[-3)-2-*O*-acetyl-*β*-D-Gal*p*-(1-4)-*α*-D-Gal*p*-(1-3)-*β*-D-Galp-(1-5)-*β*-D-KDO*p*-(2-] 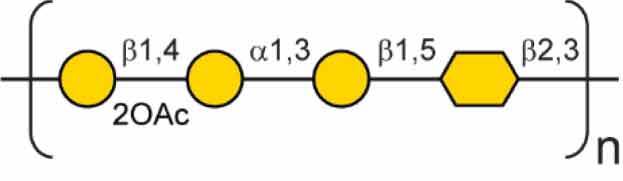	[105,106]	

6d 6-deoxy; 2OAc 2-*O*-acetyl; 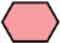 DDManHep; 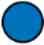 Glc; 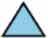 6dTal; 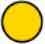 Gal; 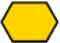 KDO.

Capsular polysaccharides (CPS) are tightly packed repeating polysaccharides that provide a barrier around bacterial cells and play a role in adhesion and pathogenicity. Four putative capsule polysaccharide (CPS) regions have been identified in *B. pseudomallei* with genes involved in sugar biosynthesis and transport (Table 1) [63,97,103]. The best characterised of these clusters is CPS I (*BPSL2787-2810*), which is present in all clinical isolates, indicating a conservation of this CPS amongst *B. pseudomallei* strains [107]. The resulting product of this biosynthetic cluster is a high molecular weight unbranched polymer of [-3)-2-*O*-acetyl-6-deoxy-*β*-D-*manno*-heptopyranose-(1-] residues, also referred to as the type I *O*-PS in some papers (Table 1) [98,103]. The CPS is thought to be a major virulence determinant, with initial studies using subtractive hybridisation, demonstrating that a CPS I-mutant had an LD_50_ similar to that of *B. thailandensis* [99]. Further work with signature-tagged transposon mutants showed that CPS mutants were unable to establish infection in mice and had a significant increase in time to death [89,108]. During infection, capsule expression is induced in the presence of serum, resulting in a decrease in levels of complement C3b deposition, thus reducing the activation of the complement cascade [100]. Deletion of the entire *wcb* capsule biosynthesis operon was successfully constructed by Warawa et al., indicating that capsule is not essential for bacterial viability. This mutant showed no significant difference from the wild-type strain when tested *in vitro* in J774A.1 murine macrophages, indicating that CPS I is not required for intracellular survival or replication of *B. pseudomallei* in a laboratory setting [109]. However, this mutant displayed a 2-log increase in LD_50_ within an intranasal model using BALB/c mice. Decreased bacterial counts in the blood and spleen were observed, alongside decreased histopathology scores in the spleen and liver. This decrease in histopathology score is thought to be due to a reduced Th1 immune response to infection, which is usually stimulated by host recognition of the CPS I [109]. Further investigations into single components of capsule biosynthesis were conducted by Cuccui et al., during which 18 of the 25 genes comprising the CPS I coding region were disrupted. Within this study, disruption of *gmhA* (*BPSL2795*), *wcbJ* (*BPSL2798*) and *wcbN* (*BPSL2793*) resulted in strains, which were unable to cause lethal disease following intranasal challenge. Immunization studies conducted with these strains prior to intranasal challenge using *B. pseudomallei* K96243 successfully increased time to death. However, immunization was not able to prevent recovery of *B. pseudomallei* from tissues of survivors, indicating that sterilizing immunity was not achieved [101].

The lipopolysaccharide (LPS) is a glycolipid molecule decorating the outside of the cell wall of Gram-negative bacteria. The LPS consists of three components, the lipid A, the core region, and an *O*-antigen polysaccharide chain (*O*-PS) [110]. The structure of the *B. pseudomallei O*-antigen was solved and found to be an unbranched heteropolymer of repeating structures of [-3)-β-D-glucopyranose-(1-3)-6-deoxy-α-L-talopyranose-(1-] and is referred to as type II *O*-PS [98,104]. The LPS biosynthesis cluster is found on Chromosome I (*BPSL2672-BPSL2688*) [63]. A comprehensive study of 1327 isolates of environmental and clinical origin was investigated to determine the heterogeneity of LPS in *B. pseudomallei* isolates. Three serotypes of LPS have been observed in *B. pseudomallei*, smooth typical type A LPS, smooth atypical type B LPS, and rough LPS. The dominant serotype is smooth type A, which accounts for 97% of all screened isolates and consists of the characterised *O*-antigen moiety, type II *O*-PS [111]. A variant of type B LPS, type B2, has now also been identified through genomic screening [112]. A Tn*5* transposon mutant of *B. pseudomallei* strain 1026b (insertion in *wbiI*, strain SRM117) deficient in type II *O*-PS was sensitive to killing in 30% non-human serum. Strain SRM117 also displayed a 10-fold reduction in virulence in both hamster and guinea-pig melioidosis models and 100-fold less virulence in an infant diabetic rat model [102]. This decrease in virulence is also seen in BALB/c mice with a 4-log increase in LD_50_ in an intraperitoneal injection model [89]. Further studies with SRM117 determined that deletion of the type II *O*-PS resulted in increased uptake into RAW264.7 murine macrophages. However, intracellular counts decreased during the early stages of infection, attributed to the activation of inducible nitric oxide synthase (iNOS), whereas macrophages infected by *B. pseudomallei* wild-type failed to activate iNOS [113]. These results indicate that the LPS, particularly the *O*-PS moiety, plays an important role in suppressing the host response during early stages of infection *in vitro* and deletion results in attenuation in animal models of disease. TLR4 is an important component of this response due to its ability to detect the LPS of Gram-negative pathogens [114]. *B. pseudomallei* can be recognised by both TLR4 and TLR2, and this has been demonstrated *in vitro*, but *in vivo* TLR2 is responsible for the detection of LPS and host response to infection [115]. Further work has shown that only upon deletion of type II *O*-PS, does an increase in TLR4-dependent NF-κB activation occur [116], perhaps explaining the *in vivo* host reliance on TLR2 activation. Differential signalling between murine and human cell line models has been shown with LPS-induced immune activation occurring solely through TLR4 within murine models both *in vitro* and *in vivo*, while additional TLR2 activation also occurs in human models [117], highlighting the importance of more research into the immune response in different models of infection.

### Phagosomal survival

Following the attachment and entry of *B. pseudomallei* into the host cell, the bacterium comes into contact with many host mechanisms that are utilised to kill and clear foreign material. Phagocytic cells employ and produce both reactive nitrogen intermediates (RNI) and reactive oxygen species (ROS) to control microbial infection [118]. *B. pseudomallei* is a weak activator of beta-interferon (IFN-β), resulting in reduced iNOS production [119]. iNOS can induce nitric oxide (NO) production and other reactive nitrogen species leading to the cytotoxic activity of macrophages against different microorganisms, intracellular parasites, and tumour cells [120]. Infection of RAW264.7 murine macrophages with *B. pseudomallei* at a MOI of 10 showed no significant expression of iNOS compared to the high production observed when infected with either *Escherichia coli* or *Salmonella typhi* at a MOI of 0.1 [121]. Furthermore, *B. pseudomallei* is susceptible to chemically generated NO in a macrophage-free system [122], highlighting the importance of the subversion of iNOS expression and blocking of the NO production for the survival of *B. pseudomallei* in macrophages.

To protect against oxidative damage, *B. pseudomallei* expresses sigma factors, RpoS, and RpoE, and the transcriptional regulator OxyR, which can directly counteract oxidative stress through their downstream effectors. Sigma factors have been shown to mediate the response of *B. pseudomallei* to oxidative stress [123]. In *B. pseudomallei*, inactivation of *rpoS* led to multiple effects including increased susceptibility to carbon starvation and oxidative stress with a *rpoS* mutant unable to induce MNGC formation [123,124]. RpoS also regulates genes involved in oxidative response including cysteine synthase B, 3-methyl-2-oxobutanoate hydroxymethyltransferase, several hypothetical stress response proteins, and succinyl-CoA: 3-ketoacid-coenzyme A transferase subunit A, with the latter shown to downregulate the expression of endogenous ROS [125]. Additionally, deletion of sigma factor RpoE gene increased the susceptibility of *B. pseudomallei* against menadione and H_2_O_2_ and showed reduced intracellular survival in J774A.1 murine macrophages [126]. Proteomic analysis of a *rpoE* mutant also showed differentially present proteins that are involved in oxidative and osmotic stress proteins in addition to chaperones, indicating the role it plays in the survival of *B. pseudomallei* under different adverse conditions [127]. The transcriptional regulator, OxyR, regulates several genes associated with oxidative stress and within *B. pseudomallei*, it exerts a bi-functional role by repressing expression of the KatG catalase under normal growth and activating it under oxidative conditions [128,129]. The expression of OxyR is further regulated by the RpoS sigma factor [130]. Inactivation of *oxyR* in *B. pseudomallei* increases the susceptibility to both H_2_O_2_ and paraquat, highlighting the essentiality of OxyR in protection against oxidative stress [128]. Taken together, this emphasises the importance of the regulatory mechanisms in the intracellular survival and oxidative resistance of *B. pseudomallei*.

There are other genes involved in protection against oxidative stress including *katG* (*BPSL2865*) which encodes for catalase-peroxidase I in *B. pseudomallei*. Deletion of *katG* increases susceptibility to killing by different oxidative agents including H_2_O_2_, menadione, *N*-ethylmaleimide (NEM), and hypochlorite [129]. Interestingly, the deletion of the *katG* gene led to increased resistance to organic hydroperoxide [131]. This was identified to be due to the upregulation of alkyl hyroperoxidase reductase encoded by the *ahpC* gene. Overproduction of AhpC was shown to be protective against both reactive oxygen species and reactive nitrogen species [131] explaining the resistance observed against *tert*-butyl hydroperoxide (*t-*BOOH) and demonstrating the role of AhpC in the intracellular survival of *B. pseudomallei*.

In addition to antioxidant effectors that can directly inactivate oxidants, *B. pseudomallei* is also armed with different proteins that can directly protect bacterial DNA. One of these proteins is the putative ferritin DPS-family DNA binding protein (*dpsA*/*BPSL2863*) encoded downstream of the *katG* gene in *B. pseudomallei*. The gene *dpsA* is regulated by both OxyR and RpoS, and under oxidative conditions, *dpsA* is co-transcribed with *katG* highlighting the multiple responses coordinated by the bacterium to resist oxidative stress [129,130]. It has been demonstrated that *dpsA* is important in the resistance of the bacterium against organic hydroperoxide with hypersensitivity to *t-*BOOH observed with the *dpsA* deletion mutant [132]. Furthermore, *B. pseudomallei* also encodes for a spermidine acetyltransferase homologue that can act as a free radical scavenger protecting the DNA [133]. This spermidine acetyltransferase, *speG*, is also indirectly regulated by RpoE under oxidative stress conditions [126]. This multi-factorial approach allows *B. pseudomallei* to survive within the lysosomes by mounting a response, detoxifying the environment, and directly protecting the bacterial DNA.

Superoxide dismutases are enzymes that are able to convert superoxides, one of the most potent reactive oxygen intermediates, into the less damaging hydrogen peroxides for better clearance by other enzymes, including KatG [129,134]. Although not shown to participate in the resistance to intracellular superoxide, superoxide dismutase C (*sodC)* of *B. pseudomallei* has been shown to provide some protection against extracellular superoxides [135]. Additionally, reduced replication in murine macrophages and attenuation in the BALB/c mice were observed upon deletion of *sodC*, demonstrating the importance of SodC in the detoxification of extracellular superoxides that may support intracellular growth of *B. pseudomallei* [135]. These studies clearly demonstrate the ability of *B. pseudomallei* to adapt to and resist the phagosomal environment by modulating gene and protein expression. However, further studies are required to fully understand the roles of sigma factors and transcriptional regulators in the virulence of *B. pseudomallei* using *in vivo* infection models.

### Phagosomal escape

*B. pseudomallei* has been shown to escape endocytic vesicles of HeLa, RAW264.7, and U937 cells within 15 minutes post-internalisation [136,137] (Figure 1). The escape of the bacterium from the phagosome before the maturation into the phagolysosome is mediated by the type III secretion system (T3SS). This enables the bacteria to escape the lysosome and enter the host cytoplasm where replication and movement into adjacent cells can occur. The T3SS resembles a molecular syringe that delivers effector proteins into host cells. Three clusters of T3SS genes have been identified in *B. pseudomallei* strain K96243 [63]. T3SS-1 of *B. pseudomallei* is homologous to the Hrp2 secretion system identified in *Ralstonia solanacearum* [138,139], whereas T3SS-2 has greater homology to the secretion systems of *Xanthomonas* spp [138,140]. T3SS-3 of *B. pseudomallei* has been characterised most extensively, and its components are named the *Burkholderia* secretion apparatus (Bsa). This secretion system has been observed to share high homology to the *inv/spa/prg* encoded T3SS of *Salmonella* Typhimurium and the *ipa/mxi/spa* T3SS of *Shigella flexneri* [141]. Several structural, translocator, and effector proteins encoded by the T3SS-3 were identified to be essential in the escape of the bacterium from phagocytic vesicles.

## Type III secretion systems (T3SS)

Deletion of genes encoding several structural proteins of the T3SS-3 has been shown to impair bacterial escape. A disruption in the *bsaZ* gene (*BPSS1534*), which encodes a structural component of the export apparatus homologous to *Salmonella* SpaS, showed a severe delay in phagosome escape as demonstrated by bacterial co-localization with the lysosomal-associated membrane protein 1 (LAMP-1) marker within J774.2 murine macrophages [141] and transmission electron microscopy of RAW264.7 murine macrophages, which showed confinement of the *bsaZ* mutant to membrane-bound compartments [142]. Insertional mutagenesis to inactivate *bsaQ* a structural component of the secretion apparatus resulted in delayed escape of the bacterium at 6 hours post-infection with the *bsaQ* mutant exhibiting greater co-localisation with LAMP-1-positive phagosomes compared to the wild-type [143]. In addition to this phenotype, the secretion of BopE and BipD was absent in the supernatant indicating the inability of the *bsaQ* mutant to secrete effector proteins. BsaU (BPSS1539) is a part of the T3SS homologous to the InvJ of *Salmonella* spp., which is responsible for the control of the needle length [144]. A *bsaU* transposon mutant showed significant impairment in vacuolar escape as visualised with LAMP-1 co-localisation staining [145]. Intracellular replication and actin tail formation were shown to be functional, but the inability of the mutant to escape the phagosomes had led to the distension of the vesicles packed with *B. pseudomallei bsaU* mutant [145].

The Bsa translocators, BipB, BipC, and BipD, sit at the tip of the Bsa needle complex forming a pore for the delivery of the bacterial effector proteins into host cells [35]. As seen with the *bsaZ* mutant, severe impairment in vacuolar escape was seen with the *bipD* mutant, including reduced invasion of HeLa human epithelial cells [146]. The *bipD* mutant was also shown to be almost exclusively co-localised with the LAMP-1-associated phagosomes in J774.2 murine macrophages, indicating the inability of the mutant to escape endocytic vesicles [141]. In addition to the severe impairment in vacuolar escape, the *bipD* mutant was also unable to induce actin rearrangements and form cellular protrusions. Delay in vacuolar escape was also observed with the inactivation of BipB and BipC, which are homologous to SipB and SipC of *Salmonella*, respectively [147,148]. Both the *bipB* and *bipC* mutant also showed reduced intracellular survival with the *bipB* mutant and also display marked reduction in MNGC formation and *in vivo* virulence [149]. This illustrates the importance of the *B. pseudomallei* Bsa translocators for escape from the phagosome and intracellular spread.

## T3SS effector proteins

In addition to the translocators, deletion of several genes that encode for effector proteins that subvert host functions has led to the delayed or impaired escape of *B. pseudomallei* in the phagosomes. BopC is an effector protein and was shown to bind to its cognate chaperone BPSS1517 [150]. Characterisation of the *bopC* mutant demonstrated reduction in intracellular survival in addition to delayed bacterial escape from the vacuoles of J774A.1 murine macrophages. Similarly, inactivation of *bopA*, another Bsa effector also resulted in reduced bacterial survival in phagosomes [151]. Increased co-localisation of the *bopA* mutant with the autophagosomal microtubule-associated protein light chain 3 (LC3) in RAW264.7 murine macrophages highlighted the impaired ability of this mutant to escape the phagosome [151]. A T3SS-3 effector protein of *B. pseudomallei, BPSS1385* (CHBP) has been shown to be a cycle inhibiting factor (Cif). Cifs are known to cause cytopathic effects in eukaryotic host cells, such as cell cycle arrest and cell death [152]. Cifs and CHBP are deamidases, which specifically deamidate the Gln40 residue on host ubiquitin and ubiquitin-like proteins [153,154]. Treatment of macrophage cells such as differentiated bone marrow-derived macrophages and J774A.1 murine macrophages with recombinant CHBP resulted in rapid cell death, while leaving epithelial cells such as HeLa cells viable [154]. Mutagenesis studies of *BPSS1385* in *B. pseudomallei* have not yet been undertaken, so its exact role *in vivo* is yet to be elucidated. Taken together, this illustrates the different functions of the T3SS and its effector proteins in facilitating bacterial escape from the endocytic vesicles as well as manipulation of host cell. The importance of the T3SS in *B. pseudomallei* also makes it an attractive target for therapeutic intervention. This was demonstrated using small molecule ATPase inhibitors, targeting the T3SS ATPase *bsaS*, which effectively reduced bacterial escape from the phagolysosome, resulting in decreased intracellular bacteria [155]. How effective these inhibitors are *in vivo* remains to be validated, however this highlights the potential for targeting the T3SS in *B. pseudomallei*.

### Intracellular adaptation and replication

Bacterial phospholipases are membrane-bound or secreted hydrolases [156]. In *B. pseudomallei*, three phospholipase C (Plc) are encoded, *plc1* (*BPSL2403*), *plc2* (*BPSL0338*), and *plc3* (*BPSS0067*) [63]. Deletion of *plc2* reduced the intracellular survival, plaque formation, and cytotoxicity in HeLa human epithelial cells, indicating that *plc2* is more associated with virulence and intracellular survival compared to *plc1* and *plc3* [157,158]. Further analysis of the *plc2* mutant showed that the reduction of intracellular survival was not due to delayed escape [158]. As mentioned previously, macrophages utilise ROS, NO, and proteases to kill internalised bacteria. For example, mature macrophages express elastase (matrix metalloproteinase 12), which enters the phagolysosomes to attach to bacterial cell walls disrupting membrane integrity and inducing bacterial cell death [159]. *B. pseudomallei* encodes the serine protease inhibitor ecotin (eco/*BPSL1054*) that is active against elastase, trypsin, and chymotrypsin [160]. This allows the bacteria to circumvent the macrophage proteolytic activity assisting intracellular survival. The deletion of ecotin resulted in reduced intracellular survival of the bacteria in J774A.1 murine macrophages and significant attenuation in the BALB/c model of infection [160]. Additionally, several ecotin orthologues were shown to inhibit the complement pathway system [161]. Taken together, it can be hypothesised that ecotin plays a role in both the inhibition of the normal macrophage degradative functions and the impairment of the immune response allowing bacterial survival.

## Essential *in vivo* metabolic pathways

**Figure 2.**
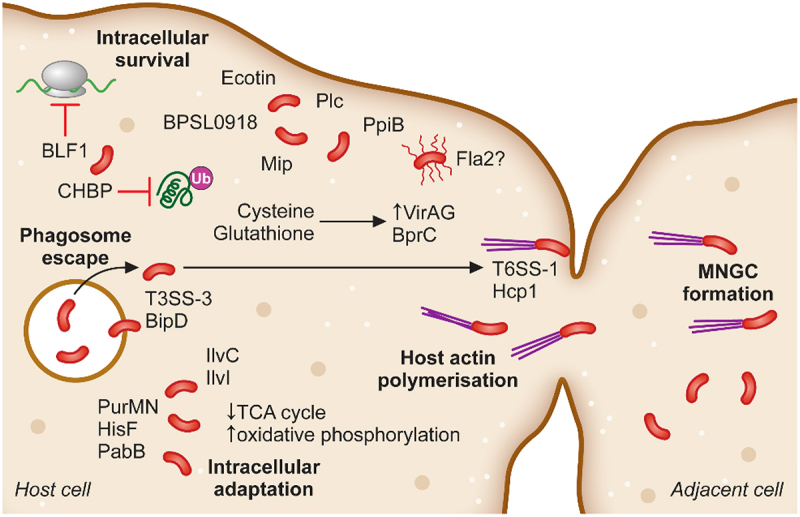
The intracellular adaptation and spread of *B. pseudomallei* from cell-to-cell. Following phagosomal escape *B. pseudomallei* switches its metabolic pathways to allow for the greatest energy production within the cytoplasmic environment. Additionally, secretion of the toxin BLF1 and the T3SS effector CHBP modulate host cell processes, halting host protein synthesis. Host cell actin is polymerised by BimAC and *B. pseudomallei* cells rapidly propel to the cellular membrane upon which their T6SS–1 is expressed and Hcp1 causes membrane fusion and the formation of multinucleated giant cells (MNGCs). MNGC formation facilitates rapid spread of bacterial infection to neighbouring cells while escaping recognition by the immune response.

Several metabolic enzymes have also been implicated to be important in the survival of the bacterium inside the host cells (Figure 2). Transposon mutagenesis has identified genes in the purine metabolism pathway to be important in intracellular survival, in particular phosphoribosylglycinamide formyltransferase (*purN/BPSL0908*) and phosphoribosylformyl-glycinamidine cyclo-ligase (*purM/BPSL2818*) [145]. The inactivation of *purN* and *purM* from *B. pseudomallei* strain E8 resulted in decreased intracellular survival within HeLa cells, with the latter showing no intracellular replication. Furthermore, gene deletions that affected the histidine (*hisF/BPSL3133*) and para-aminobenzoate biosynthetic pathways (*pabB/BPSL2825*) also exhibited reduced intracellular survival in HeLa cells [145]. The reason proposed in the study is that the metabolic intermediates of these biosynthetic pathways are limited inside the host cells, and therefore, any further disruption via mutagenesis leads to impaired intracellular survival and replication. Additionally, a deletion of *asd* (*BPSS1704*), which encodes the enzyme aspartate-β-semialdehyde dehydrogenase, important for amino acid biosynthesis, resulted in the generation of a diaminopimelate (DAP) auxotrophic strain that was unable to grow in rich media without supplementation and displayed reduced intracellular replication in both RAW264.7 murine macrophages and HeLa cells [162]. Like other metabolic mutants, the absence of DAP in mammalian cells thus becomes detrimental to the survival of the auxotrophic bacterium in cells [162].

Other genes encoding for enzymes that participate in amino acid biosynthesis are equally important for *B. pseudomallei* survival and virulence. In particular, enzymes involved in the biosynthesis of the branched chain amino acids (BCAAs) leucine, isoleucine, and valine have been found to be essential for virulence. Disruption of *ilvI* (*BPSL1196*), encoding an acetolactate synthase catalytic subunit involved in 2-oxocarboxylic acid metabolism, was first discovered incidentally and investigated further due to the loss of virulence in a murine melioidosis model. The *ilvI* transposon mutant was auxotrophic for leucine, isoleucine, and valine. This gene assists the conversion of both pyruvate and 2-oxobutanoate into intermediate metabolites that form the skeleton for these BCAAs. The transposon mutant was unable to persist within mouse models or cause lethal infections to the same extent as wild-type *B. pseudomallei* strain 576. Interestingly, this gene is non-essential in rich media, but downstream components of the BCAA biosynthesis are necessary for survival (*ilvC/BPSL1198*) [163,164].

## Energy metabolism during *in vivo* infection

Transcriptomic studies of *B. pseudomallei* following infection of Syrian hamsters showed an upregulation of genes involved in the intermediate steps of the glycolytic pathway (phosphoglycerate mutase, *BPSL2902*), inositol catabolism (*BPSL1996, BPSL1997*), glucosamine catabolism (*glmS3*), and tryptophan catabolism/synthesis (*trpE, asnO, glmS3*). Interestingly, genes involved in oxidative phosphorylation such as *cyoA, cyoB*, and *fdsB*, were highly induced, while genes involved in the TCA cycle (*fumC, sucD* and *phbB*), serine catabolism/synthesis (*glyA, BPSL2219*), and energy metabolism (ATP-synthase, NADH-dehydrogenase) were highly down regulated [165]. The switch to alternative energy sources may be an adaptation for acute infection, which the Syrian hamster model represents, as inhibition of the TCA cycle enzyme, isocitrate lyase (*aceA/BPSL2188*), in Sprague-Dawley rats (a chronic model of melioidosis), results in the switching of infection from a persistent state to a lethal acute infection if untreated by antibiotics [166]. This points to *B. pseudomallei* being metabolically flexible and able to utilise alternate energy sources during infection *in vivo*.

## Toxins and their regulation

Toxins have also been shown to play a role in *B. pseudomallei* infection. The gene, *BPSL1549*, encodes for a toxin or Burkholderia Lethal Factor 1 (BLF1), which shows homology to *E. coli* toxin cytotoxic necrotizing factor 1 (CNF1) deamidase. This toxin is upregulated during virulence cues such as in the presence of human serum, taurine, and insulin. Purified BPSL1549 caused cell toxicity to J774.2 murine macrophages after 72 hours and a mutant strain, *B. pseudomallei ΔBPSL1549* was severely attenuated in BALB/c mice and displayed a 100-fold increase in median lethal dose. BPSL1549 was shown to bind to the eukaryotic transcription initiation factor, eIF4A. BPSL1549 causes deamidation of Gln339 of eIF4A, which results in potent inhibition of host translation and protein synthesis [167]. A putative toxin regulatory gene, *BPSL1527* or *tex*, codes for a transcription accessory protein, which has been shown to be involved in toxin regulation in other pathogens. A deletion mutant, *Δtex* displayed reduced CFU numbers in spleen and lungs 48 hours post-infection and decreased survival in A549 epithelial cells and J774A.1 murine macrophages [168]. It is not yet known if *tex* is directly involved in toxin regulation in *B. pseudomallei* as studies to determine this have not yet been published.

## Folding proteins

Two members of the immunophilin superfamily, *BPSS1823* (*mip*) and *BPSL0918*, have also been identified as important for intracellular survival of *B. pseudomallei* in J774A.1 murine macrophages [169,170]. These proteins are peptidyl-prolyl *cis-trans* isomerases and are involved in protein folding and chaperone activity [171]. In addition, deletion of *mip* resulted in reduced motility as well as protease production and attenuation in a BALB/c mouse model [170]. The proteins are likely to be playing a critical role in the folding of virulence factors required for *B. pseudomallei* to establish intracellular infection.

### Cell-to-cell spread

#### Host actin polymerisation

*B. pseudomallei* is able to propel itself within a host cell by polymerising host cell actin [172,173] (Figure 2). This is primarily mediated by the protein *Burkholderia* intracellular motility A (BimA), which was inferred through homology to other auto-secreted proteins in pathogens that polymerise host cell actin, in particular the YadA protein of *Yersinia enterocolitica* [173]. Deletion of *bimA* results in loss of actin-based motility as shown in infected J774.2 murine macrophages, where bacterial cells cluster in the cytoplasm but exhibit a distinct lack of polar actin tails and cellular protrusions [173]. Using monoclonal antibodies against BimA, Stevens et al. demonstrated that BimA localises to a singular pole of the bacterial cell with an F-actin tail occurring from the same pole. The polymerisation of actin into these actin tails is thought to propel *B. pseudomallei* towards the host cell membrane and cause cellular protrusion from which cell-to-cell spread is then mediated [173]. BimA from *B. pseudomallei* is capable of polymerising actin *in vitro* independent of the Arp (actin-related protein) 2/3 complex, the protein complex that regulates and nucleates eukaryotic actin [173]. However, the Arp 2/3 complex, as well as α-actinin, was found within the actin tail polymerised by BimA activity [174]. Interestingly, recent work has shown that BimC, encoded directly upstream of *bimA* in the genome, is also required for actin tail formation in HeLa cells, but this deficiency was only partially restored by complementation [175]. Although polar localisation of BimA still occurred in the *ΔbimC*-mutant strain, actin tail formation was absent. This indicates that BimC participates to some extent in BimA-mediated actin polymerisation [175].

## Multinucleated giant cell formation (MNGC)

Following the movement of *B. pseudomallei* through the cytoplasm, fusion of the host cell membranes occurs and MNGCs are formed (Figure 2). This phenomenon has also been demonstrated in bone biopsies from human infections as well as in post-mortems [176], indicating that this biological process occurs both within the laboratory and during natural infection. *B. pseudomallei*-infected RAW264.7 murine macrophages are induced to form MNGCs during infection with increased expression of calcitonin receptor (CTR), cathespin K (CTSK), and tartrate-resistant acid phosphatase (TRAP), markers which are hallmarks of functional osteoclasts, natural MNGCs formed from the fusion of mononuclear cells [177,178], although further work showed that *B. pseudomallei*-induced MNGCs are only osteoclast-like [177]. Studies showed that *lfpA* (lactonase family protein A; *BPSS2074*) was responsible for the increase in expression of osteoblast markers, CTR, CTSK, and TRAP seen in RAW264.7 murine macrophages. Additionally, *lfpA* is required for optimal virulence in animal models, as deletion of *IfpA* results in a 4.5-fold-decrease in LD_50_ and a delayed time-to-death in Syrian hamsters and BALB/c mice [177]. The formation of MNGCs by the host has been attributed to the type VI secretion system (T6SS) of *B. pseudomallei*, in particular the Cluster 1 T6SS [179].

## Type VI secretion systems (T6SS)

T6SS are essential components for virulence in a variety of pathogens including *Vibrio cholerae* and *Francisella tularensis* [180,181], as they are contact-dependent secretion apparatuses, which can inject toxins and other effectors into eukaryotic cells [182]. *B. pseudomallei* encodes six T6SS clusters termed clusters 1 through to 6 (Table 2). There are currently two nomenclatures for these T6SS based on work by Schell et al. and Shalom et al. which were both published in 2007. This review will use the Schell et al. nomenclature whereby clusters 1-6 are as follows: T6SS-1 (*tss-5*), T6SS-2 (*tss-4*), T6SS-3 (*tss-6*), T6SS-4 (*tss-3*), T6SS-5 (*tss-2*), and T6SS-6 (*tss-1*) (Table 2) [190,191]. These clusters share high levels of homology with those found in *B. mallei* and *B. thailandensis*, but interestingly, T6SS-4 is absent in *B. thailandensis* and T6SS-5 is absent in *B. mallei*, while T6SS-6 is truncated in *B. mallei* [190] indicating that there may be a specific role for these clusters in *B. pseudomallei*. Expression of T6SS in *B. pseudomallei* is tightly controlled, with only T6SS-6 showing detectable levels of expression in nutrient broth [179], with further studies demonstrating a 12-fold increase in expression of T6SS-1 following invasion into macrophages [191]. Deletion of the inner tubule of the contractile sheath, composed of polymerised hexameric haemolysin-coregulated protein (Hcp) rings, of each T6SS cluster determined that only deletion of *hcp1* of T6SS-1 was essential for disease in the Syrian Golden hamster model [179], indicating that T6SS-1 is the major virulence-associated T6SS of *B. pseudomallei*. This mutant, *Δhcp1*, also displayed decreased intracellular counts and was less cytotoxic to RAW264.7 murine macrophages. This was corroborated by fluorescence microscopy, which showed a defect in intracellular spread with the inability to form MNGCs [179]. Hcp proteins, in addition to being a structural component of the T6SS, can act as chaperones for effector proteins, although further experimental work needs to be conducted to determine this in *B. pseudomallei* [192,193]. Toesca et al. also demonstrated that the tip of the T6SS needle, VgrG, is also required for MNGC formation, in particular the C-terminal domain, which mediates host membrane fusion [183] and that T6SS-1 is localised to the bacterial cell pole [184]. T6SS-1 in *B. pseudomallei* was recently reviewed by Lennings et al. [194].

**Table 2.** **Type VI secretion systems (T6SS) of *B. pseudomallei***. *B. pseudomallei* encodes for six T6SS clusters as determined by homology to cluster of orthologous genes (COG) in other Gram-negative pathogens. Two nomenclature systems for *B. pseudomallei* T6SS were published in 2007, Schell et al. and Shalom et al. [190,191]. Both nomenclatures are listed as well as the known functions of these clusters in *B. pseudomallei*.

*Strain K96243 gene codes*	Schell et al. nomenclature	Shalom et al. nomenclature	Function/notes	Ref
*BPSS1496-BPSS1511*	T6SS-1	*tss-5*	Essential for virulence. Located on the pole of the bacterium. Deletion of Hcp1, VgrG results in loss of MNGC formation. Expression is induced by cysteine and reduced glutathione.	[179,183–185]
*BPSS0515-BPSS0533*	T6SS-2	*tss-4*	Negatively regulated by TctR. Induced under oxidative stress conditions. Repressed by OxyR. Exports metal chelators to increase zinc and manganese into the bacterial cell.	[186–189]
*BPSS2090-BPSS2109*	T6SS-3	*tss-6*		
*BPSS0166-BPSS0185*	T6SS-4	*tss-3*	Absent in *B. thailandensis.*	
*BPSS0091-BPSS0117*	T6SS-5	*tss-2*	Absent in *B. mallei.*	
*BPSL3096-BPSL3111*	T6SS-6	*tss-1*	Contact-dependent bacterial competition. Unable to persist in mixed biofilms with *P. putida*. Positively regulated by TctR.	[184,186]

Activation of the T6SS-1 is mediated by the VirAG two-component system, which senses host intracellular signals, thus activating transcription of genes encoding components of the T6SS-1 such as *hcp1, tssAB*, and *vgrG*. The T3SS regulator protein, BprC, mediates expression of *tssAB* but not of *hcp1* and *virAG* [195]. Work by Wong et al. determined that exposure of *B. pseudomallei* to cysteine and reduced glutathione, present in the cytoplasm of host cells, activated the two-component system sensor kinase VirA, driving expression of T6SS-1 [185]. Expression of T6SS-1 components is detectable at 3 hours post-infection and increases until 6 hours post-infection [195].

The precise roles of the remaining five T6SS clusters requires further investigation, with only Cluster 1 shown to be essential for virulence in the mouse model [179]. Studies in *B. thailandensis* have shown that T6SS-6 is important for contact-dependent bacterial competition, with deletion of *clpV* (encoding an AAA+ ATPase required for recycling the contracted sheath), resulting in a strain unable to persist in mixed population biofilms with *Pseudomonas putida* [186]. A MarR transcription factor (TctR) was found to negatively regulate the expression of T6SS-2 but positively regulates T6SS-6, but *ΔtctR* was indistinguishable from wild-type in a Syrian hamster infection model [187], indicating that TctR does not play a role in regulating the T6SS-1, which is essential for disease. T6SS-2 is expressed under oxidative stress conditions in *B. thailandensis* and has been observed to be regulated by OxyR. Overexpression of T6SS-2 was observed in the *B. thailandensis ΔoxyR* mutant [188]. This strain is also required for export of zinc and manganese chelators into the extracellular space for combating oxidative stress [189,196]. These studies indicate that each of the T6SSs in *B. pseudomallei* may be important for survival in different environmental niches, investigation into this has been limited to date, with focus mainly on the intracellular environment.

Another protein important for *B. pseudomallei* intracellular spread and MNGC formation is the cyclophilin *ppiB*. The *ppiB* gene is essential for establishment of disease in BALB/c mice [197]. It was shown that deletion of *ppiB* resulted in major disruption of the bacterial proteome ultimately leading to a 67% reduction in MNGC formation [197], likely due to disruption of T6SS assembly *in vitro* and *in vivo*, although further work needs to be done to corroborate this.

In conclusion, *B. pseudomallei* has maintained numerous strategies to successfully establish a niche in humans and animals. *B. pseudomallei* employs many mechanisms for cell entry, vacuole escape, and subsequent cell-to-cell spread. It subverts the immune response to allow for bacterial proliferation, spreads to all systems in the host causing a multitude of symptoms, and can rapidly develop into septic pneumonia which can be fatal.

### Author’s perspective

*Burkholderia pseudomallei* remains a resilient environmental pathogen capable of causing major morbidity in tropical regions of the world. Although studies have now unravelled mechanisms by which *B. pseudomallei* infects and replicates in the host, there is still much left to understand, such as its ability to persist, hide away effectively, and then remerge to cause infection years later. Unique mechanisms for virulence in *B. pseudomallei*, such as Arp2/3-independent actin polymerisation by BimA [173] and multiple functional homologues of virulence factors such as the autotransporters, *boaA* and *boaB* [82], mean careful manipulation and characterisation is required to fully understand the molecular mechanisms of pathogenicity. Additionally, different studies suggest variable roles of classical virulence factors. An example is the flagellum, in *B. pseudomallei* KHW strain, the absence of the flagella results in attenuation in BALB/c mice [86]; in contrast, in strain 1026b, loss of flagella had no impact on the virulence using the Syrian hamster model [88]. These instances highlight the differences between strains and infection models used between different laboratories to investigate these molecular mechanisms. Furthermore, only a small fraction of the genome has been investigated using genetic and biochemical methods, although high-throughput techniques such as transposon insertion site sequencing (TraDIS) have revealed a number of genes important for pathogenesis. To fully understand the pathogenicity of *B. pseudomallei*, more investment into genetic tools and characterisation of genes important for pathogenesis and persistence is required. With no currently licensed vaccine and treatment compliance moderate at best, particularly in endemic areas, more work is needed to find novel intervention points, which could be used in conjunction with current antibiotic therapies. This review has highlighted many virulence factors and lifestyle adaptations, which are required by *B. pseudomallei* to cause infection and survive within the host. Selection of some of these virulence determinants for therapeutic intervention studies, with the focus on synthesis of novel inhibitors, should support the development of new strategies to help melioidosis patients and those living in endemic areas.
